# Validation of a Brief Internet-Based Self-Report Measure of Maladaptive Personality and Interpersonal Schema: Confirmatory Factor Analysis

**DOI:** 10.2196/48425

**Published:** 2023-09-29

**Authors:** Hyeonseong Kim, Seohyun Jeong, Inae Hwang, Kiyoung Sung, Woori Moon, Min-Sup Shin

**Affiliations:** 1 Psychiatry Department Seoul National University Hospital Seoul Republic of Korea; 2 40FY Inc Seoul Republic of Korea; 3 Seoul National University College of Medicine Seoul Republic of Korea

**Keywords:** maladaptive schema, measure of schema, self-report measure, internet-based measure, digital mental health care, interpersonal schema

## Abstract

**Background:**

Existing digital mental health interventions mainly focus on the symptoms of specific mental disorders, but do not focus on Maladaptive Personalities and Interpersonal Schemas (MPISs). As an initial step toward considering personalities and schemas in intervention programs, there is a need for the development of tools for measuring core personality traits and interpersonal schemas known to cause psychological discomfort among potential users of digital mental health interventions. Thus, the MPIS was developed.

**Objective:**

The objectives of this study are to validate the MPIS by comparing 2 models of the MPIS factor structure and to understand the characteristics of the MPIS by assessing its correlations with other measures.

**Methods:**

Data were collected from 234 participants who were using web-based community sites in South Korea, including university students, graduate students, working professionals, and homemakers. All the data were gathered through web-based surveys. Confirmatory factor analysis was used to compare a single-factor model with a 5-factor model. Reliability and correlation analyses with other scales were performed.

**Results:**

The results of confirmatory factor analysis indicated that the 5-factor model (*χ*^2^_550_=1278.1; Tucker-Lewis index=0.80; comparative fit index=0.81; and Root Mean Square Error of Approximation=0.07) was more suitable than the single-factor model (*χ*^2^_560_=2341.5; Tucker-Lewis index=0.52; comparative fit index=0.54; and Root Mean Square Error of Approximation=0.11) for measuring maladaptive personality traits and interpersonal relationship patterns. The internal consistency of each factor of the MPIS was good (Cronbach α=.71-.88), and the correlations with existing measures were statistically significant. The MPIS is a validated 35-item tool for measuring 5 essential personality traits and interpersonal schemas in adults aged 18-39 years.

**Conclusions:**

This study introduced the MPIS, a concise and effective questionnaire capable of measuring maladaptive personality traits and interpersonal relationship schemas. Through analysis, the MPIS was shown to reliably assess these psychological constructs and validate them. Its web-based accessibility and reduced item count make it a valuable tool for mental health assessment. Future applications include its integration into digital mental health care services, allowing easy web-based administration and aiding in the classification of psychological therapy programs based on the obtained results.

**Trial Registration:**

ClinicalTrials.gov NCT05952063; https://www.clinicaltrials.gov/study/NCT05952063

## Introduction

Digital mental health services include mental health care programs provided via web or mobile platforms, thereby extending traditional face-to-face mental health care. These services use web-based platforms, offering advantages such as cost savings and improved accessibility. Digital mental health services have been rapidly growing in the recent years because of these benefits [1]. Many studies have reported their effectiveness in treating a range of mental disorders such as obsessive-compulsive disorder, depression, panic disorder, insomnia, and attention-deficit/hyperactivity disorder [2,3]. However, digital mental health services that are currently reported to be effective, mostly focus on the treatment of specific mental disorders or syndromes, thus making it difficult to expand their scope to enhance the mental health of the general population. Thus, meeting the needs of adults reporting a diverse range of psychological discomfort, ranging from subclinical symptoms to personality issues, is still a challenge.

Alternatively, interventions that focus on maladaptive personality traits and coping styles should be considered in the field of relevant research [4]. Psychological discomfort varies depending on how individuals identify and respond to stress [5]. Personality traits refer to an individual’s tendencies to think and act consistently or similarly in different situations, and certain personality traits can become risk factors for stress in specific situations [6]. For instance, a trait that places significant importance on anxiety and exhibits avoidance behaviors can trigger stress in an individual more easily in situations like an examination or a presentation.

To provide interventions based on maladaptive personality traits within digital mental health services, it is necessary to conduct a selection process for representative types of maladaptive personality traits. In that regard, a review of prior research is essential to determine how maladaptive personality traits can be categorized. The Diagnostic and Statistical Manual of Mental Disorders, 5th edition (DSM-5) [7] identifies five broad trait domains: (1) negative affectivity, (2) detachment, (3) antagonism, (4) disinhibition, and (5) psychoticism, as maladaptive variants corresponding to the traits presented in the 5-factor model of personality. Each trait domain is further divided into 25 specific trait facets, including insecurity, hostility, withdrawal, and rigid perfectionism. Additionally, some researchers have considered early maladaptive schemas, a key concept in schema therapy, as maladaptive personality traits [8]. Early maladaptive schemas are defined as self-defeating patterns of emotion and thought that begin early in an individual’s life and are repeated throughout their life [9]. It comprises five broad categories: (1) disconnection and rejection, (2) impaired autonomy and performance, (3) impaired limits, (4) other-directedness, and (5) overvigilance and inhibition, along with 18 psychological schemas under subcategories like emotional deprivation, dependence or incompetence, entitlement or grandiosity, self-sacrifice, and unrelenting standards or hypercriticalness [10].

Due to the diversity of maladaptive personality traits, digital mental health care services seeking to intervene must specify and evaluate the maladaptive personality traits that the intervention seeks to mitigate. It is important to note that intervention and evaluation must be conducted in a non–face-to-face environment. However, current personality traits and schema assessment scales may not be well-suited for on the internet use due to their length and large number of items, which makes them unsuitable for web-based implementation and thereby increases the entry barriers to intervention programs at the initial assessment phase. For example, the Personality Inventory for DSM-5 (PID-5), which assesses the personality traits of the alternative model of DSM-5 personality disorders, presented earlier, comprises 220 items [11], and the Young Schema Questionnaire-Long form-3 (YSQ-L3), which is mainly used to evaluate early maladaptive schemas, is composed of 232 items [9]. Furthermore, an intuitive tool is essential for web-based interventions, as users must independently recognize the need for intervention through an initial assessment and evaluate the perceived effectiveness of the program by checking the trend of self-reported scores before deciding whether to continue. However, it is difficult for potential users of digital mental health services to intuitively understand their psychological characteristics without an expert’s assistance when using existing scales. For example, there are 25 trait facets in PID-5, and it is difficult to understand each trait facet without an expert explanation. Furthermore, it is difficult to intuitively understand various maladaptive personality patterns depending on whether the level of each facet is high or low, and a combination of it. While in schema therapy, psychological schemas may be explored through additional interviews instead of relying solely on results of the YSQ-L3 [9]. Therefore, considering the potential implications for digital mental health services, a valid assessment scale that can evaluate an individual’s maladaptive psychological characteristics on the internet in a concise and intuitive manner is critical.

For this study, the Maladaptive Personality and Interpersonal Schema (MPIS; 40FY Inc, unpublished), which is a brief internet-based self-report measure, was developed as a way to measure maladaptive psychological characteristics and schemas of individuals. Input from 1 psychiatrist and 3 clinical psychologists, examination of various existing scales, and consultation with experts on item development, reliability, and validity were crucial steps in developing the scale. In the process of scale development, 1 psychiatrist and 3 clinical psychology experts generated 50 items. Subsequently, correlation analysis and exploratory factor analysis were conducted among the items, leading to the final selection of 35 items for the MPIS. Exploratory factor analyses were conducted, and five primary psychological schemas that have the potential to induce stress were identified as follows: (1) Shelly (social isolation), related to lack of belongingness to a group and social skills; (2) Flippy (anger), related to a lack of patience and hot-tempered coping style; (3) Riggy (perfectionism), related to strict standards; (4) Pleaser (low self-esteem), related to a tendency to excessively pursue affection and attention from others; and (5) Jumpy (anxiety), related to pessimistic and anxious tendencies. Through web-based survey responses, participants could identify which maladaptive psychological characteristics and schemas they aligned with and also obtain information about the severity of those characteristics and schemas. This study thus verified the reliability and validity of the MPIS.

## Methods

### Recruitment and Survey Procedure

This study recruited adult participants from large web-based community sites. The website used for participant recruitment was a community site open to a diverse range of occupations and age groups, including university students, graduate students, working professionals, and homemakers. The recruitment period was from July 26, 2022, to August 16, 2022.

This study used a cross-sectional design with a convenience sample. The inclusion criteria were as follows: (1) 18 to 39 years of age; (2) the ability to read and write in Korean without external assistance; and (3) the ability to access websites and respond to questions using a mobile phone or computer. Furthermore, individuals who refused to complete the survey without a specific reason or deliberately gave inappropriate responses repeatedly during their participation were excluded from this study. A 2-step method was used to detect careless responses [12]. First, the ratio of the number of identical responses to the total number of items for each participant was calculated, and the top 5% (12/234) of participants with the highest repetition of the identical responses were selected. Second, an analysis was conducted to determine whether these participants' responses appeared as straight-line patterns within their respective questionnaires. If straight linings were not detected, their responses were included in the final analysis.

Instructions on how to complete and submit this study’s participation application on the internet were provided in the form of a post on the internet community site. Participants who completed this study’s participation application received explanations about the participation and consent form completion process through phone calls or emails. Additionally, participants were sent an email containing a link to access the web-based survey. Once participants submitted their final responses, the answers were automatically saved on the researchers' computer server.

The target participation size for conducting factor analysis was determined to be at least 5 times the number of MPIS items, which amounted to 175 individuals [13]. In internet-based survey research, participant dropout rates have been observed to vary widely, typically ranging from 10% to 30% [14]. Additionally, the rate of careless responses varies from 0.5% to 14% [12]. Adopting a conservative perspective, the rate of dropout and careless responses was assumed to be 40% (70/175). As a result, the final target number of participants was set at 250 individuals.

Participants voluntarily completed a study consent form and then took part in a 30-minute web-based survey. Survey items were presented in a predetermined order to prevent response bias. Additionally, all surveys displayed only 2-3 questions per screen, and as participants completed their responses, the screen would automatically scroll to the next set of questions. Each page of the survey included a maximum of 45 items, and the survey consisted of a total of 15 pages. A completeness check was implemented for each item, requiring participants to answer all questions on a page before proceeding to the next page. Furthermore, a review step was provided to allow participants to review and modify their answers before submitting their responses. To identify unique visitors, only 1 response per participant was collected using an assigned URL for each participant. Prior to participant recruitment, technical tests were conducted to ensure the proper functioning of the web-based survey.

The informed consent form specified the survey duration, types of data collected, data retention period, investigators, research objectives, scope of personal information collection, and anonymization methods for ensuring personal information protection. As compensation for their participation, participants received a monetary reward of 10,000 KRW (equivalent to US $7 as of August 2023) and the MPIS results report.

### Measures

#### Measure of MPIS

The MPIS comprises 35 items rated on a 5-point Likert scale, with higher scores indicating a greater presence of dysfunctional psychological personality traits. In the process of scale development, the internal consistency (Cronbach α) of the scale was .86 for Shelly, .86 for Flippy, .78 for Riggy, .74 for Pleaser, and .85 for Jumpy.

#### Perceived Stress Scale

The Perceived Stress Scale (PSS) [15,16] comprises 10 items rated on a 4-point Likert scale, with 6 items assessing negative perceptions and 4 assessing positive ones. The items reflecting positive perceptions are reverse scored with a higher overall score indicating a higher degree of perceived stress. The PSS assesses the extent to which individuals perceive life events as stressful and experience unpredictability, uncontrollability, and excessive demands in daily life. The negative and positive perceptions were found to be positively correlated with depression, anxiety, and negative emotions, and negatively correlated with positive emotions. In this study, the internal consistency was 0.87 for negative perceptions and 0.73 for positive perceptions.

#### Self-Efficacy Scale

The Self-Efficacy Scale (SES) [17,18] has 17 items related to achievement self-efficacy and 6 related to social self-efficacy, rated on a 5-point Likert scale. Higher scores indicate a higher level of self-efficacy. In this study, the internal consistency was 0.93 for achievement self-efficacy and 0.75 for social self-efficacy.

#### Center for Epidemiologic Studies Depression Scale

The Center for Epidemiologic Studies Depression Scale (CES-D) [19,20] comprises 20 items rated on a 4-point Likert scale, measuring depression experienced by individuals over the past week. In this study, the internal consistency of the scale was found to be 0.93.

#### State-Trait Anxiety Inventory

The State-Trait Anxiety Inventory (STAI) [21] comprises 20 items for state anxiety and 20 for trait anxiety on a 4-point Likert scale. In this study, 20 items related to trait anxiety were used. The internal consistency in this study was 0.92 for trait anxiety.

#### University of California-Los Angeles Loneliness Scale

The University of California-Los Angeles Loneliness Scale (UCLA-LS) [22,23] comprises 20 items rated on a 4-point Likert scale that asks about social relationships in a positive or negative direction. Items in the positive direction were reverse scored and summed with a higher score indicating a higher degree of loneliness. In this study, the internal consistency was 0.94.

#### Short Form of the Korean Inventory of Interpersonal Problems Circumplex Scale

The short form of the Korean Inventory of Interpersonal Problems Circumplex Scale (KIIP-SC) [24] comprises 40 items rated on a 5-point Likert scale, 18 of which address topics associated with having difficulties relating to others and 22 of which describe situations where it seems like things are “too much.” Subfactors are Korean Inventory of Interpersonal Problems domineering or controlling, Korean Inventory of Interpersonal Problems vindicative or self-centered, Korean Inventory of Interpersonal Problems cold or distant, Korean Inventory of Interpersonal Problems socially inhibited or avoidant (KIIP-FG), Korean Inventory of Interpersonal Problems nonassertive (KIIP-HI), Korean Inventory of Interpersonal Problems overly accommodating or exploitable, Korean Inventory of Interpersonal Problems self-sacrificing or overly nurturant (KIIP-LM), and Korean Inventory of Interpersonal Problems intrusive or needy (KIIP-NO). In this study, the overall internal consistency was 0.93, and the internal consistency for each subfactor was 0.71 (Korean Inventory of Interpersonal Problems domineering or controlling), 0.81 (Korean Inventory of Interpersonal Problems vindicative or self-centered), 0.85 (Korean Inventory of Interpersonal Problems cold or distant), 0.88 (KIIP-FG), 0.88 (KIIP-HI), 0.80 (Korean Inventory of Interpersonal Problems overly accommodating or exploitable), 0.68 (KIIP-LM), and 0.66 (KIIP-NO).

#### Diagnostic Test for Personality Disorders-Dependent Subscale

The Diagnostic Test for Personality Disorders-Dependent subscale (DTPD-D) [25] is a 15-item instrument rated on a 4-point Likert scale that measures dependent personality disorder, as defined in the DSM-IV, and its predisposition. In this study, the internal consistency was 0.86.

#### State-Trait Anger Expression Inventory

The State-Trait Anger Expression Inventory (STAXI) [26,27] comprises 20 items for state and trait anger and 24 items for anger expression style (anger-in, anger-out, and anger-control) on a 4-point Likert scale. In this study, 24 items related to anger expression style were used. The internal consistency in this study was 0.82 for anger-in, 0.82 for anger-out, and 0.60 for anger-control.

#### Impulsive Behavior Scale

The Urgency-Premeditation-Perseverance-Sensation seeking -Positive urgency [28-30] is a 59-item measure, rated on a 4-point Likert scale with five subfactors: (1) negative urgency, (2) lack of premeditation, (3) lack of perseverance, (4) sensation seeking, and (5) positive urgency. Only items belonging to the negative urgency category were used for this study. In the present sample, the overall internal consistency was 0.95, and the internal consistency for the negative urgency factor was 0.89.

#### Hewitt Multidimensional Perfectionism Scale

The Hewitt Multidimensional Perfectionism Scale (HMPS) [18,31] comprises 45 items rated on a 7-point Likert scale with three subfactors: (1) self-oriented perfectionism, (2) other-oriented perfectionism, and (3) socially prescribed perfectionism. In this study, the internal consistency was 0.92 for self-oriented perfectionism, 0.82 for other-oriented perfectionism, and 0.85 for socially prescribed perfectionism.

#### State Self-Esteem Scale

The State Self-Esteem Scale (SSES) [32,33] is a 20-item measure, with 7 items on performance self-esteem, 6 on appearance self-esteem (referred to as “general self-esteem” in the Korean version), and 7 on social self-esteem, rated on a 5-point Likert scale. A higher score is associated with higher self-esteem. In this study, internal consistency was 0.87 for performance self-esteem, 0.81 for appearance self-esteem, and 0.86 for social self-esteem.

#### Rejection Sensitivity Questionnaire

The Rejection Sensitivity Questionnaire (RSQ) [34,35] comprises 18 situations in which an individual makes demands of a significant other, such as a parent, friend, or lover in daily life. In each situation, the anxiety associated with rejection of one’s request (rejection anxiety) and the expectation that the other person will accept it (acceptance expectation) were evaluated on a 6-point Likert scale. Items corresponding to acceptance expectations were reverse scored and interpreted as rejection expectations. In this study, the internal consistency was 0.93.

#### Beck Anxiety Inventory

The Beck Anxiety Inventory (BAI) [36,37] is a 21-item measure for evaluating anxiety severity on a 4-point Likert scale. A total score of 0-7 corresponds to normal anxiety, 8-15 to mild anxiety, 16-25 to moderate anxiety, and 26-63 to high anxiety. The internal consistency in this study was 0.95.

#### Intolerance of Uncertainty Scale

The Intolerance of Uncertainty Scale (IUS) [38-40] is a 12-item measure rated on a 4-point Likert scale. In this study, the internal consistency was 0.87.

#### Penn State Worry Questionnaire

The Penn State Worry Questionnaire (PSWQ) [41,42] is a 16-item instrument rated on a 5-point Likert scale. It measures the frequency and intensity of chronic, uncontrollable worry. In this study, the internal consistency was 0.94.

### Data Analysis

Data for this study were analyzed using the following processes. First, confirmatory factor analysis (CFA) was conducted using the Mplus (version 7; Muthén & Muthén) for Windows. A valid model was established by comparing the goodness of fit indices of the hypothesized 5-factor model with the alternative single-factor model. The estimation of model parameters was conducted using the maximum likelihood method. To evaluate the goodness of fit of the model, the chi-square, Tucker-Lewis index (TLI), comparative fit index (CFI), and root-mean-square error of approximation (RMSEA) values were reported. The cutoff used for the factor loading to remove any item from the MPIS was 0.4 [13]. Even when the factor loading of an item was below 0.4, the final decision on including the item in the MPIS was based on comparing model fit indices and information indices between models with and without the item, and considering the item's content. In addition, internal consistency was assessed using Cronbach α. Convergent and discriminant validity were then evaluated using the Pearson correlation coefficient.

### Ethical Considerations

All study procedures were approved by the institutional review board of Seoul National University Hospital (IRB H-2203-108-1309).

## Results

### Characteristics of Participants

A total of 323 individuals applied to participate in this study, and after excluding those who withdrew from the survey, a total of 250 participants successfully completed the web-based survey. The completion rate was 77% (250/323). A total of 16 participants who were outside the age range were excluded. The analysis was conducted only on the completed questionnaires. There was no participant who met the criteria for careless response, and all 234 samples were included in the analysis. The participants had a mean age of 26.33 (SD 5.41) years. Table 1 presents the demographic information.

**Table 1:** Demographic information (N=234).

		Value, n (%)
**Sex**		
	Male	77 (32.9)
	Female	157 (67.1)
**Age (years)**		
	18-29	160 (68.4)
	30-39	74 (31.6)
**Marital status**		
	Unmarried	195 (83.3)
	Married	39 (16.7)
**Education (years)**		
	12	97 (41.5)
	14	4 (1.7)
	16	133 (56.8)

### Characteristics of Each MPIS Item

In Table 2, the mean range of each MPIS item was 2.07 to 4.01, and the SD range of each item was 0.87 to 1.39. For all items, the absolute values of skewness and kurtosis did not exceed 2 and 7, respectively. The result is provided in Multimedia Appendix 1. Thus, it was assumed that the data followed a normal distribution [43].

**Table 2:** Factor loading of the MPIS^a^ (N=234).

Item number	Shelly (factor 1)	Flippy (factor 2)	Riggy (factor 3)	Pleaser (factor 4)	Jumpy (factor 5)	Mean (SD)
29	0.78	— ^b^	—	—	—	2.52 (1.18)
35	0.77	—	—	—	—	2.43 (1.26)
19	0.74	—	—	—	—	2.66 (1.30)
25	0.71	—	—	—	—	2.91 (1.27)
10	0.70	—	—	—	—	2.21 (1.07)
17	0.70	—	—	—	—	2.71 (1.16)
1	0.66	—	—	—	—	2.49 (1.09)
2	0.64	—	—	—	—	2.81 (1.21)
24	0.54	—	—	—	—	2.64 (1.33)
11	0.36	—	—	—	—	2.94 (1.17)
16	—	0.86	—	—	—	2.65 (1.24)
9	—	0.79	—	—	—	2.59 (1.14)
23	—	0.79	—	—	—	2.53 (1.11)
5	—	0.72	—	—	—	2.20 (1.17)
28	—	0.65	—	—	—	2.46 (1.19)
31	—	0.59	—	—	—	2.07 (1.12)
33	—	—	0.78	—	—	3.29 (1.08)
27	—	—	0.68	—	—	3.67 (1.13)
14	—	—	0.67	—	—	2.94 (1.16)
7	—	—	0.67	—	—	4.01 (0.87)
22	—	—	0.66	—	—	3.60 (1.11)
21	—	—	—	0.80	—	2.53 (1.16)
26	—	—	—	0.64	—	2.85 (1.22)
32	—	—	—	0.64	—	3.19 (1.15)
13	—	—	—	0.55	—	2.43 (1.31)
18	—	—	—	0.54	—	2.41 (1.19)
6	—	—	—	0.04	—	3.41 (1.04)
15	—	—	—	—	0.73	3.26 (1.27)
4	—	—	—	—	0.72	2.50 (1.19)
8	—	—	—	—	0.71	3.18 (1.17)
34	—	—	—	—	0.71	3.44 (1.14)
12	—	—	—	—	0.70	3.12 (1.39)
3	—	—	—	—	0.61	2.50 (1.16)
30	—	—	—	—	0.57	3.07 (1.21)
20	—	—	—	—	0.53	2.51 (1.32)

^a^MPIS: Maladaptive Personality and Interpersonal Schema.

^b^—: not available.

### Confirmatory Factor Analysis

To validate the MPIS, which was predefined, a CFA was conducted. The proposed model consisted of 5 factors. Meanwhile, an alternative model was a single-factor structure.

Regarding the results of the CFA for the single-factor model, the model fit indices are unacceptable (*χ*^2^_560_=2341.5; TLI=0.52; CFI=0.54; and RMSEA=0.11). However, for the results of the CFA for the 5-factor model, the model fit indices were acceptable, or slightly less than the good fit values (*χ*^2^_550_=1278.1; TLI=0.80; CFI=0.81; and RMSEA=0.07). Therefore, the conclusion drawn was that the 5-factor model was more suitable than the single-factor model.

In Table 2, the factor loading for item 6 was 0.04, which is below 0.40. There is a need for consideration regarding the exclusion of item 6. With the exception of item 6, the revised 5-factor model displayed model fit indices that are acceptable, or slightly less than the criteria for a good fit (*χ*^2^_517_=1196.5; TLI=0.81; CFI=0.82; and RMSEA=0.07). This is not significantly different from the proposed model that includes item 6.

Additionally, information criteria were examined when comparing the proposed model and the revised 5-factor model. The proposed model yielded Akaike Information Criterion=22,890.95 and Bayesian Information Criterion=23,288.31, while the revised 5-factor model showed Akaike Information Criterion=22,201.73 and Bayesian Information Criterion=22,588.73, indicating minimal discrepancy between the 2 models. Given that item 6 encompassed essential content for the composition of the MPIS (a question regarding perceived interpersonal schemas in individuals with low self-esteem), it was retained as part of the final item set for the scale. The path model of 5-factor model is described in Multimedia Appendix 2.

### Reliability

First, Cronbach α was .88 for factor 1, .87 for factor 2, .82 for factor 3, .71 for factor 4 (but increased to .77 when item 6 was excluded), and .86 for factor 5. Looking at the range of correlation coefficients between the subitems and the total scores by factor, it was 0.48-0.77 for factor 1, 0.70-0.85 for factor 2, 0.73-0.80 for factor 3, 0.59-0.78 for factor 4 (correlation coefficient between item 6 and factor 4 total score was 0.28), and 0.63-0.75 for factor 5.

### Convergent and Discriminant Validity

In this study, PSS, SES, CES-D, and State-Trait Anxiety Inventory were used because the dysfunctional schema is associated with high stress levels, low self-efficacy, and high negative emotions such as depression and anxiety. Furthermore, the UCLA-LS, KIIP-SC, DTPD-D, STAXI, Urgency-Premeditation-Perseverance-Sensation seeking-Positive urgency Impulsive Behavior Scale-Negative Urgency, HMPS, SSES, RSQ, BAI, IUS, and PSWQ were used to verify the validity of the measure’s subfactors.

Tables 3 and 4 present the results of the analyses. First, the correlation between the measure’s subfactors was in the range of 0.17-0.63 (median 0.38; *P*<.001), indicating that the subfactors were appropriately differentiated. The total score of the MPIS was negatively correlated with SES, and positively correlated with PSS, CES-D, and State-Trait Anxiety Inventory.

Consequent to the correlation analysis of the subfactors and reference scale, Shelly was positively correlated with UCLA-LS, KIIP-FG, and DTPD-D. Flippy was positively correlated with STAXI anger-in, anger-out, anger-control, and Urgency-Premeditation-Perseverance-Sensation seeking-Positive urgency Impulsive Behavior Scale-Negative Urgency. Riggy was positively correlated with HMPS self-oriented perfectionism, other-oriented perfectionism, and socially prescribed perfectionism. Pleaser showed a negative correlation with SSES-social and a positive correlation with KIIP-HI, KIIP-LM, KIIP-NO, RSQ-rejection anxiety, and RSQ-rejection expectation. Jumpy showed a positive correlation with BAI, IUS, and PSWQ. These results demonstrate that the MPIS has good convergent and discriminant validity.

**Table 3:** Mean, SD, and Pearson correlation coefficients of factor-wise total scores for the MPIS^a^ (N=234).

	Mean (SD)	Pearson correlation coefficient				
		Shelly (factor 1)	Flippy (factor 2)	Riggy (factor 3)	Pleaser (factor 4)	Jumpy (factor 5)
Shelly	26.54 (8.39)	—^b^	—	—	—	—
Flippy	14.49 (5.51)	0.38	—	—	—	—
Riggy	17.52 (4.12)	0.37	0.17	—	—	—
Pleaser	16.82 (4.56)	0.45	0.27	0.34	—	—
Jumpy	23.59 (7.07)	0.63	0.39	0.51	0.51	—

^a^MPIS: Maladaptive Personality and Interpersonal Schema.

^b^—: not available.

**Table 4:** Pearson correlation coefficients between the MPIS^a^ and related assessment (N=234). A high score for SES^b^ means high self-efficacy. A high score for SSES^c^ means a higher state of self-esteem.

MPIS and related assessment		Pearson correlation coefficient
**Total composite score**		
	PSS^d^	0.65
	SES	–0.52
	CES-D^e^	0.64
	STAI^f^	0.74
**Shelly**		
	UCLA-LS^g^	0.73
	KIIP-FG^h^	0.65
	DTPD-D^i^	0.46
**Flippy**		
	STAXI^j^ anger-in	0.41
	STAXI anger-out	0.60
	STAXI anger-control	0.45
	UPPS-NU^k^	0.63
**Riggy**		
	HMPS^l^ self-oriented	0.60
	HMPS other-oriented	0.21
	HMPS socially prescribed	0.30
**Pleaser**		
	SSES-social	–0.47
	KIIP-HI^m^	0.52
	KIIP-LM^n^	0.57
	KIIP-NO^o^	0.31
	RSQ^p^-rejection anxiety	0.36
	RSQ-rejection expectation	0.18
**Jumpy**		
	BAI^q^	0.55
	IUS^r^	0.60
	PSWQ^s^	0.79

^a^MPIS: Maladaptive Personality and Interpersonal Schema.

^b^SES: Self-Efficacy Scale.

^c^SSES: State Self-Esteem Scale.

^d^PSS: Perceived Stress Scale.

^e^CES-D: Center for Epidemiologic Studies Depression scale.

^f^STAI: State-Trait Anxiety Inventory.

^g^UCLA-LS: University of California - Los Angeles Loneliness Scale.

^h^KIIP-FG: Korea Inventory of Interpersonal Problems circumplex scale-socially inhibited or avoidant.

^i^DTPD-D: Diagnostic Test for Personality Disorders-Dependent.

^j^STAXI: State-Trait Anger Expression Inventory.

^k^UPPS-NU: Urgency-Premeditation-Perseverance-Sensation seeking-Positive urgency impulsive behavior scale-Negative Urgency.

^l^HMPS: Hewitt Multidimensional Perfectionism Scale.

^m^KIIP-HI: Korean Inventory of Interpersonal Problems nonassertive.

^n^KIIP-LM: Korean Inventory of Interpersonal Problems self-sacrificing or overly nurturant.

^o^KIIP-NO: Korean Inventory of Interpersonal Problems intrusive or needy.

^p^RSQ: Rejection Sensitivity Questionnaire.

^q^BAI: Beck Anxiety Inventory.

^r^IUS: Intolerance of Uncertainty Scale.

^s^PSWQ: Penn State Worry Questionnaire.

## Discussion

### Principal Findings

This study verified the reliability and validity of an internet-based self-report measure developed to assess MPIS. First, the result of the CFA indicating that the 5-factor model is more suitable than the single-factor model signifies that understanding maladaptive personality traits and interpersonal relationship patterns measured by the MPIS in 5 distinct types is justified. Particularly, the significance of these findings lies in the prospect of using the MPIS as a screening and classification measure for future digital mental health care services. This is enabled by deriving scores for each factor through the MPIS and thereby offering type-specific psychological therapy programs to individuals with high severity scores within each factor.

Next, item 6 had a low factor loading. While this might suggest a possibility of low validity of the item, it cannot be excluded that the interaction between item content and respondent characteristics also played a role. Those who excessively attribute importance to the interest or approval of others tend to adapt their behavior to gain such attention or recognition. Item 6, respondents were questioned about their efforts to satisfy the needs of others. However, even individuals excessively valuing the attention or recognition of others might not be fully aware of their behavioral patterns or could psychologically deny them. These possible causes might have contributed to the low factor loading observed for item 6.

Finally, based on the analysis of convergent and discriminant validity, the MPIS factors were all found to be appropriately distinct. Additionally, it was indicated that each factor adequately measures the targeted maladaptive personality and interpersonal relationship schema.

The implications of this study are as follows. First, a reliable and valid measure that can effectively identify an individual’s maladaptive personality traits was obtained. Previous measures like the PID-5 and YSQ-L3 identified 25 and 18 personality traits or psychological schemas, respectively, using more than 200 items [9,11]. Contrastingly, the MPIS uses only 35 items derived from the 5 core personality traits associated with individual maladjustment, which is more suited to digital mental health interventions as it allows participants to complete the questionnaire in a short time. Another benefit of the measure is that it is available on the internet. Currently, psychological measures conducted offline have not been easy for respondents to access; therefore, they are often underused to meet the needs of people who want to be accurately evaluated for their schema or who seek to establish therapeutic interventions based on the respondent's schema. However, on the internet, availability of these measures can improve usability and motivation to participate by increasing public accessibility. Finally, this measure may be useful for preventing the exacerbation of psychological problems.

### Limitations

This study has some limitations. First, the majority of the sample comprised undergraduate and graduate students (133/234, 56.8%). Recruitment was conducted through community sites for office workers, stay-at-home spouses, and so on to diversify the participants; however, in the analysis, the student group still comprised the majority. Due to the nature of web-based surveys administered through platforms like mobile apps and computers, it seems that the higher accessibility for younger student age groups could be attributed to their ample personal usage time for smartphones or computers. In future research, validation could be conducted by considering the characteristics of the actual population group and implementing sampling to ensure diversity within the sample.

Second, the measures used may have underestimated some statistical results such as reliability due to an insufficient number of items corresponding to each maladaptive personality trait. In particular, some personality traits were measured using only 5 items, so the number of items per factor in the measure was disproportionate. It is difficult to detect maladaptive personality traits which are abstract psychological concepts using a small number of items. Therefore, effort is needed to add items without compromising the simplicity of the measure.

Third, item 6, which showed a low factor loading in the factor analysis, was determined to be appropriate and was not excluded from the measure, but future research should consider excluding item 6 or replacing it with another new item, thus considering the correlation of items by factor.

Fourth, individual personality traits were evaluated using only self-reported measures. There is no experimental approach to observe the actual behavioral patterns of individuals to analyze how much they correspond to the results of the maladaptive personality traits tests. In subsequent studies, it may be possible to construct scenarios or experimental situations related to anxiety, perfectionism, anger, low self-esteem, and isolation. This could help analyze whether maladaptive personality traits and interpersonal relationship schemas measured by the MPIS are correlated with descriptive responses or actual behaviors.

Fifth, in this study, only the single-factor model and the 5-factor model were compared using CFA, and a broader range of models was not incorporated into the analysis. Subsequent research could encompass a variety of models based on theoretical foundations beyond just the single-factor and 5-factor models.

### Conclusions

This study has resulted in the development of a questionnaire capable of measuring 5 types of maladaptive personality traits and interpersonal relationship schemas. Through analysis, it was established that the 5 factors effectively measure psychological traits. The noteworthy significance of the MPIS lies in its web-based administration, providing excellent accessibility, cost-effectiveness, and featuring a reduced number of items compared to traditional psychological scales.

In the future, the MPIS could prove instrumental in offering digital mental health care services. Its web-based administration facilitates easy access, allowing for the convenient implementation of the MPIS and enabling the categorization of psychological therapy program content based on the results.

## Appendix

Multimedia Appendix 1 Skewness and kurtosis of the Maladaptive Personality and Interpersonal Schema.

Multimedia Appendix 2 The path model of the five-factor model.

## Abbreviations

BAI: Beck Anxiety Inventory
CES-D: Center for Epidemiologic Studies Depression Scale
CFA: confirmatory factor analysis
CFI: comparative fit index
DSM-5: Diagnostic and Statistical Manual of Mental Disorders, 5th edition
DTPD-D: Diagnostic Test for Personality Disorders-Dependent
HMPS: Hewitt Multidimensional Perfectionism Scale
IUS: Intolerance of Uncertainty Scale
KIIP-FG: Korean Inventory of Interpersonal Problems socially inhibited or avoidant
KIIP-HI: Korean Inventory of Interpersonal Problems nonassertive
KIIP-LM: Korean Inventory of Interpersonal Problems self-sacrificing or overly nurturant
KIIP-NO: Korean Inventory of Interpersonal Problems intrusive or needy
KIIP-SC: Korean Inventory of Interpersonal Problems Circumplex Scale
MPIS: Maladaptive Personality and Interpersonal Schema
PID-5: Personality Inventory for Diagnostic and Statistical Manual of Mental Disorders, 5th edition
PSS: Perceived Stress Scale
PSWQ: Penn State Worry Questionnaire
RMSEA: Root Mean Square Error of Approximation
RSQ: Rejection Sensitivity Questionnaire
SES: Self-Efficacy Scale
SSES: State Self-Esteem Scale
STAXI: State-Trait Anger Expression Inventory
TLI: Tucker-Lewis index
UCLA-LS: University of California - Los Angeles Loneliness Scale
YSQ-L3: Young Schema Questionnaire-Long form-3

## Data Availability

The data sets analyzed during this study are available from the corresponding author on reasonable request.
