# Supplementary material for: Validation of a Brief Internet-Based Self-Report Measure of Maladaptive Personality and Interpersonal Schema: Confirmatory Factor Analysis
Source: Interact J Med Res. 2023 Sep 29;12:e48425. doi: 10.2196/48425 (PMC10576229; doi:10.2196/48425)
Supplement: Multimedia Appendix 1 [file ijmr_v12i1e48425_app1.docx]

| Item no. | Skewness | | Kurtosis | |
| --- | --- | --- | --- | --- |
|  | Statistics | standard error | Statistics | standard error |
| 1 | .53 | .15 | -.60 | .31 |
| 2 | -.01 | .15 | -1.21 | .31 |
| 3 | .48 | .15 | -.68 | .31 |
| 4 | .33 | .15 | -1.01 | .31 |
| 5 | .67 | .15 | -.62 | .31 |
| 6 | -.26 | .15 | -.68 | .31 |
| 7 | -.99 | .15 | .95 | .31 |
| 8 | -.23 | .15 | -1.03 | .31 |
| 9 | .28 | .15 | -.89 | .31 |
| 10 | .71 | .15 | -.17 | .31 |
| 11 | -.01 | .15 | -.98 | .31 |
| 12 | -.21 | .15 | -1.28 | .31 |
| 13 | .37 | .15 | -1.26 | .31 |
| 14 | .00 | .15 | -1.01 | .31 |
| 15 | -.33 | .15 | -1.06 | .31 |
| 16 | .16 | .15 | -1.18 | .31 |
| 17 | .22 | .15 | -.92 | .31 |
| 18 | .48 | .15 | -.90 | .31 |
| 19 | .29 | .15 | -1.09 | .31 |
| 20 | .37 | .15 | -1.16 | .31 |
| 21 | .31 | .15 | -.98 | .31 |
| 22 | -.58 | .15 | -.53 | .31 |
| 23 | .47 | .15 | -.71 | .31 |
| 24 | .28 | .15 | -1.23 | .31 |
| 25 | -.05 | .15 | -1.13 | .31 |
| 26 | -.02 | .15 | -1.15 | .31 |
| 27 | -.81 | .15 | -.07 | .31 |
| 28 | .50 | .15 | -.77 | .31 |
| 29 | .40 | .15 | -.88 | .31 |
| 30 | -.22 | .15 | -1.09 | .31 |
| 31 | .80 | .15 | -.49 | .31 |
| 32 | -.34 | .15 | -.89 | .31 |
| 33 | -.18 | .15 | -.72 | .31 |
| 34 | -.51 | .15 | -.71 | .31 |
| 35 | .45 | .15 | -.98 | .31 |
