# Supplementary figures and images for: Validation of a Brief Internet-Based Self-Report Measure of Maladaptive Personality and Interpersonal Schema: Confirmatory Factor Analysis
Source: Interact J Med Res. 2023 Sep 29;12:e48425. doi: 10.2196/48425 (PMC10576229; doi:10.2196/48425)

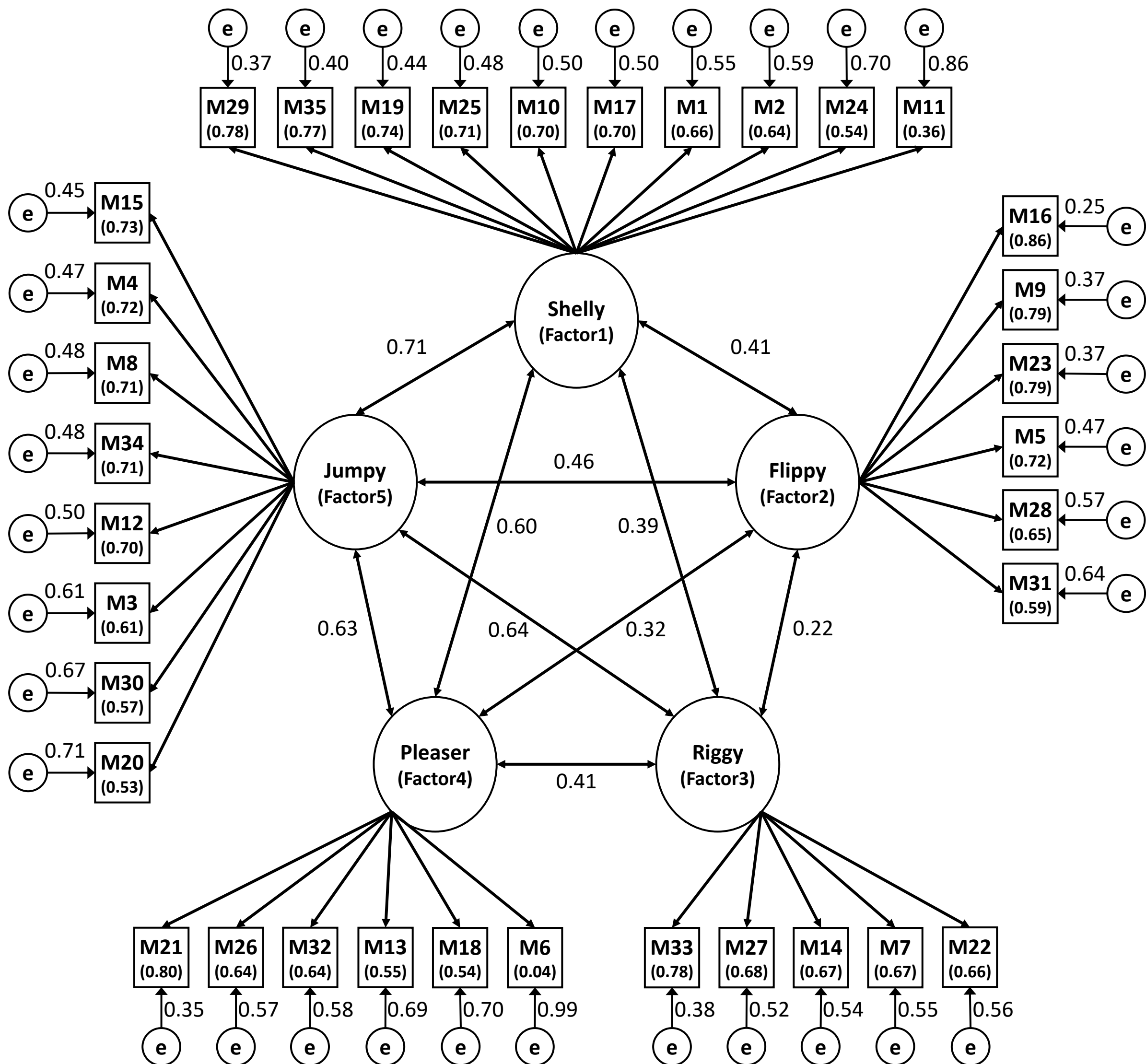

Supplement: Multimedia Appendix 2 [file ijmr_v12i1e48425_app2.pdf]
